# Oral Health and Feto-maternal Outcomes in the Context of Sustainable Development Goals

**DOI:** 10.7759/cureus.3500

**Published:** 2018-10-26

**Authors:** Sunali Khanna, Sharvari S Khedkar, Shalini Malhotra

**Affiliations:** 1 Internal Medicine, Nair Hospital Dental College, Mumbai, IND; 2 Obstetrics and Gynecology, Al Qassimi Hospital, Sharjah, ARE

**Keywords:** oral health, maternal health, sustainable development

## Abstract

The linkages of oral health with the holistic health of an individual are now well-established. Nevertheless, marginalizing oral health continues to pose a challenge in the public health scenario globally and especially in developing countries. Maternal and fetal health have been regarded as pivotal yardsticks of development in all civil societies. The oral cavity acts as a route of entry for various microorganisms into the body and oral lesions are easily detectable. Thus, early intervention is possible and morbidity in the vulnerable populations can be reduced.

## Introduction and background

Sustainable Development Goals (SDGs) became official on January 1, 2016, and the deadline set for their achievement is 2030. The health goal is framed to fit broad terms that are relevant to all countries and all populations. They are intended to be inclusive of all populations, with economic, social, and environmental sustainable development being the core focus.

Health holds a central place in Sustainable Development Goal (SDG) No. 3, that is, “To ensure healthy lives and promote well-being for all at all ages.” As there cannot be health without oral health, it becomes an integral part of SDGs. The implications of poor oral health during the vulnerable state of pregnancy are known to dental surgeons and gynecologists. Despite the awareness, no rule of the thumb is prescribed for the management of women suffering from oral conditions. Previous surveys suggest that almost 85% of gynecologists are aware of the consequences of periodontal disease on the outcome of pregnancy but still the rate of referral remains low for oral health screening [1-2].

Globally, various steps have been taken to reduce the disparities and to establish health equality among populations. However, the vulnerable populations, such as women and children, keep suffering due to lack of resources. Childbirth continues to take a toll on many females and is a prime reason for increased mortality in this population [3]. It is known that the Millennial Development Goals have paved the way for SDGs in 2016; however, it is startling to know that the impact of oral health, direct or indirect, on one’s lifestyle is not considered even in the SDGs [4]. Thus, there is a need to contemplate on how we can integrate oral health promotion and oral health care with other sectors that influence health by using the common risk factor approach [5]. It is essential for the health-related global agendas to widen to emphasize oral health issues.

The oral cavity is not an exception when the body undergoes various physiological changes. Increased or altered hormonal secretion and fetal growth induce several systemic changes, which collectively make dental treatment more challenging than routine treatment [4]. Also, procedures that can be done are at times questionable and, hence, the apprehension among pregnant patients and dentists needs to be addressed. Unfortunately, even the wide range of pain medications available is at times insufficient to cure certain types of pain, leading to an inability to control pain and thus causing the patients to suffer. Pain relief has acquired the status of a human right at a global level, to overcome the hurdles against effective pain control. Health professionals encounter these problems daily and must have knowledge of pain control to ensure efficient and safe pain treatment [6]. Moreover, women should be assured about the safety of analgesics and risks of treating versus not treating pain during pregnancy and lactation [7].

## Review

Oral health in pregnancy

Gingivitis, gingival hyperplasia, pyogenic granuloma, and salivary alterations are some of the common mucosal conditions occurring in pregnancy [8]. Erosion and dental caries are commonly occurring conditions affecting the hard tissue. They occur particularly due to an increase in the acidic environment of the oral cavity because of a decrease in salivary pH, severe vomiting, and dietary changes [9]. Melasma, appearing as bilateral brown patches in the mid-face, is seen in above 70% of pregnant women.

Periodontitis and Gingivitis

Recent studies have associated periodontal disease with pregnancy. Estrogen levels increase the capillary permeability, thus predisposing pregnant women to gingivitis and gingival hyperplasia. According to the American Academy of Periodontology, above 50% of women experience pregnancy gingivitis, which is characterized by bleeding and swollen gums [10]. Pregnancy gingivitis is the inflammation of gums caused by plaque accumulation and reduced host response. Periodontitis is an inflammatory condition, involving both the gingiva and the alveolar bone, that worsens during pregnancy. Gram-negative anaerobic organisms produce various chemical mediators such as interleukins, prostaglandins, and the tumor necrotic factor which are responsible for the destruction of periodontal tissue. Various studies on the alteration in salivary pH have proven that the salivary pH reduces progressively till the third trimester [11]. As a result, there is an increased caries incidence as well as a rise in mucosal lesions with over 44% pregnant women suffering from them [11].

Most of the studies have highlighted preterm/low birth weight as the consequence of periodontitis [10]. Studies have suggested a substantial association of periodontitis and pre-eclampsia in non-smokers [10]. The infected periodontal tissues may be associated with prematurity by acting as reservoirs of bacteria and their products, which can disseminate to the fetus -placenta unit. Furthermore, high concentrations of immunological mediators produced locally at the infected gingival tissues or systemically can reach the fetus-placenta unit, resulting in a low birth weight. Although some aspects of the association between periodontitis and complications in pregnancy are elucidated, this relationship needs to be better studied and characterized.

Caries Burden

Caries is considered an irreversible microbial disease affecting the hard tissues, which leads to cavitation, causing sensitivity and pain. Delaying the treatment of dentinal caries in the first or third trimester can have serious effects, as dentinal caries may progress to pulpitis. Space infection, pulp necrosis, dentoalveolar abscesses are some of the consequences of untreated pulpitis. Hence, to avoid/restrict these, the prompt treatment of caries is necessary.

The post-natal transmission of caries-causing bacteria, such as Streptococcus mutans, from mother to child occurs through common practices such as sharing of spoons, contaminated pacifiers, and even kissing the child. The capacity of the child to harbor these bacteria depends on the diet, oral hygiene practices, and fluoride exposure [12]. A recent study by Shearer et al. attempts to correlate maternal oral health with caries experience in the adulthood of the child [12].

Dental Erosion

Dental erosion is classified based on its cause as intrinsic and extrinsic. Extrinsic factors are acidic fruits, aerated drinks, etc. An increase in intra-abdominal pressure during pregnancy may result in increased reflux. Dental erosion is rare unless vomiting is chronic, as in the case of hyperemesis gravidarum.

Pyogenic Granuloma

Pyogenic granuloma, also known as pregnancy tumor, is a nonspecific growth that occurs frequently in pregnant women. The treatment plan depends on the severity of its interference with normal function. Conservative management, such as plaque control, is preferred over surgical methods. However, surgical removal in case of a hindrance to mastication or continuous trauma to the growth helps to curb secondary infection [13]. Even though recurrence is often seen, the lesion subsides by itself postpartum.

Temporomandibular Disorders (TMDs)

Temporomandibular joint (TMJ) problems significantly cause discomfort and affect activities of daily living. It has shown serious effects on patients such as sleep disturbances, irritability, reduced physical activity, anxiety, and depression [14]. During pregnancy, certain factors such as sleep disruption, morning sickness, and increased relaxin secretion contribute to temporomandibular joint disorders (TMDs). Other important factors, such as impaired occlusion and muscle function, are other causative factors. Studies in the past have also revealed a correlation between TMDs and Vitamin D levels [15]. Vitamin D insufficiency, as well as deficiency, is higher in females due to social customs that restrict women from being exposed to direct sunlight [16]. Even though it is considered a self- limiting disorder, certain measures can be helpful to reduce the suffering. Avoiding certain food items and particular habits that induce TMD can curtail the impact of TMD to an extent. Appliances such as oral appliances or bite plates have a cushioning effect on the TMJ and relieve the jaw muscles. Night guards, exercises for jaw muscles, and some relaxation methods have shown to improve TMDs [14].

Safety of Analgesics

They are the primary medicines used to treat various types of pain. Ineffective pain control disturbs the patient physically as well as mentally. Thus, prescribing an adequate but safe drug dose is necessary.

Traditionally, paracetamol is considered the safest of all the analgesics due to the lowest side effects. Non-steroidal anti-inflammatory drugs (NSAIDs) are avoided, as they are inhibitors of cyclooxygenase and cause the premature closure of the ductus arteriosus and pulmonary resistance vessel [17]. Opioids such as codeine are commonly found in over-the-counter drugs. Other alternatives are considered before opting for opioids [7]. Most of the analgesics are compatible the during lactation period [18].

Fortunately, the commonly used drugs in dentistry are safe in pregnancy. Nevertheless, dentists must evaluate carefully the risks versus the benefits of prescribing or administering any drug to a pregnant patient. As always, it is important to note that in a patient with pain or infection, the first line of treatment should be the removal of its source. If pharmacotherapy is initiated, prescribing the lowest dose for the shortest duration is ideal. The United States Food and Drug Administration (US FDA) has graded the documentation of risk of causing birth defects into five categories. According to these categories, Category A and B drugs can be prescribed during pregnancy (Table 1) [19].

**Table 1:** Common drugs used in dentistry

Generic Name	FDA Category	Recommended Usage
Paracetamol	B	First drug of choice for dental pain in pregnancy
Ibuprofen	B (D in 3^rd^ trimester)	Avoided completely in the 3^rd^ trimester
Diclofenac	C	Contraindicated
Metronidazole	B	Contraindicated in the 1^st^ trimester. Considered safe in the 2^nd^ and 3^rd^ trimesters
Penicillins +/- Beta-lactamase inhibitor	B	Considered safest antibiotics in pregnancy
Cephalosporins	B	As an alternative in case of allergy to amoxicillin
Erythromycin	B	Second choice of drug to penicillins
Vitamins	A	Safe in all trimesters

## Conclusions

For mankind to utilize the vast expanse of natural resources and sustain it over a long period, it is imperative for professionals to work towards the successful realization of SDGs. Therefore, engaging everyone becomes the key to fruitful implementation and actualization. Moreover, academic institutions need to advocate the training of oral health professionals to develop an educational strategy for SDG training and education. To achieve universal health coverage outcomes, it is important to improve geographical coverage of health services, provide higher coverage of skilled birth attendance, and have a smaller rich-poor disparity. This, in turn, includes a universal coverage of maternal and child health services. Addressing food security, malnutrition, environmental safety, universal health coverage, and maternal and child health requires multidimensional and multisectoral policy interventions.
